# Mapping the SRS-22r questionnaire onto the EQ-5D-5L utility score in patients with adolescent idiopathic scoliosis

**DOI:** 10.1371/journal.pone.0175847

**Published:** 2017-04-17

**Authors:** Carlos King Ho Wong, Prudence Wing Hang Cheung, Dino Samartzis, Keith Dip-Kei Luk, Kenneth M. C. Cheung, Cindy Lo Kuen Lam, Jason Pui Yin Cheung

**Affiliations:** 1 Department of Family Medicine and Primary Care, The University of Hong Kong, Hong Kong SAR, China; 2 Department of Orthopaedics and Traumatology, The University of Hong Kong, Pokfulam, Hong Kong, SAR, China; Seoul National University College of Medicine, REPUBLIC OF KOREA

## Abstract

This is a prospective study to establish prediction models that map the refined Scoliosis Research Society 22-item (SRS-22r) onto EuroQoL-5 dimension 5-level (EQ-5D-5L) utility scores in adolescent idiopathic scoliosis (AIS) patients. Comparison of treatment outcomes in AIS can be determined by cost-utility analysis. However, the mainstay spine-specific health-related quality of life outcome measure, the SRS-22r questionnaire does not provide utility assessment. In this study, AIS patients were prospectively recruited to complete both the EQ-5D-5L and SRS-22r questionnaires by trained interviewers. Ordinary least squares regression was undertaken to develop mapping models, which the validity and robustness were assessed by using the 10-fold cross-validation procedure. EQ-5D-5L utility scores were regressed on demographics, Cobb angle, curve types, treatment modalities, and five domains of the SRS-22r questionnaire. Three models were developed using stepwise selection method. EQ-5D-5L scores were regressed on 1) main effects of SRS-22r subscale scores, 2) as per 1 plus squared and interaction terms, and 3) as per 2 plus demographic and clinical characteristics. Model goodness-of-fit was assessed using R-square, adjusted R-square, and information criteria; whereas the predictive performance was evaluated using root mean square error (RMSE), mean absolute error (MAE), and the proportion of absolute error within the threshold of 0.05 and 0.10. A total of 227 AIS patients with mean age of 15.6 years were recruited. The EQ-5D-5L scores were predicted by four domains of SRS-22r (main effects of ‘Function’, ‘Pain’, ‘Appearance’ and ‘Mental Health’, and squared term of ‘Function’ and ‘Pain’), and Cobb angle in Model 3 with the best goodness-of-fit (R-square/adjusted R-square: 62.1%/60.9%). Three models demonstrated an acceptance predictive performance in error analysis applying 10-fold cross-validation to three models where RMSE and MAE were between 0.063–0.065 and between 0.039–0.044, respectively. Model 3 was therefore recommended out of three mapping models established in this paper. To our knowledge, this is the first study to map a spine-specific health-related quality of life measure onto EQ-5D-5L for AIS patients. With the consideration and incorporation of demographic and clinical characteristics, over 60% variance explained by mapping model 3 enabled the satisfactory prediction of EQ-5D-5L utility scores from existing SRS-22r data for health economic appraisal of different treatment options.

## Introduction

Adolescent idiopathic scoliosis (AIS) is the most common form of scoliosis, representing structural curves detected during adolescence without any clear underlying cause.[1] This type of scoliosis accounts for, up to 80% of idiopathic scoliosis in the United States, in otherwise healthy individuals undergoing puberty.[2] These patients often experience truncal imbalance, cosmetic unsightliness and even back pain with associated reduction in physical and mental health.[3–5] In severe cases, cardiopulmonary compromise may occur.[6, 7] These presentations in addition to curve progression may lead to a worsened impact on patients’ health-related quality of life (HRQoL) as a whole. The latest version of Scoliosis Research Society (SRS) instruments, the Refined Scoliosis Research Society 22-item (SRS-22r) questionnaire[8], is adopted to capture the HRQoL of AIS patients. The SRS-22r has been shown as a valid and reliable tool which has been used extensively over the past decade. From a recent systematic review of studies reporting quality of life, the most frequently used instruments were Scoliosis Research Society 22-item, 30-item and 24-item (SRS-22, SRS-30, SRS-24) questionnaires, contributing to approximately three-fourth of the reviewed studies.[9]

For AIS management, bracing is usually offered for moderate-sized progressive curves and surgery is offered for severe curvatures of over 40–45°.[4, 10] With increased disease prevalence and economic burden imposed to the healthcare system, evidence regarding cost-utility becomes an essential component for critical appraisal of available treatment options. Among those with curves between 35° to 55°, there is controversy upon which whether observation, bracing or surgery is better indicated in the context of cost-utility or HRQoL.[11, 12] Differentiating treatment options such as surgical approaches, implant strategy and instrumentation systems with regards to cost-utility is also necessary. As such, little is known to elucidate the health economic values of AIS treatment modalities.

As utility score data is an essential input for quality-adjusted life-years (QALYs) calculation in cost-utility analysis,[13] there is a need to develop an algorithm that maps HRQoL scores from scoliosis-specific instrument onto utility scores. Despite the good correlation between SRS-22 and generic 36-Item Short Form Health Survey (SF-36) questionnaires, the existing mapping model available for predicting SRS-22 subscale scores from SF-36 subscale scores for AIS patients is in the mental health and pain domains only.[14] In search of a more appropriate measure specifically for AIS, the EuroQol-5 Dimension (EQ-5D) utility score has been used in all economic evaluation submitted to health technology assessment authorities such as the National Institute for Health and Care Excellence (NICE) in UK.[15] This goes beyond ethnic and cultural background as the EuroQol-5 Dimension 5-Level (EQ-5D-5L) utility score has been shown to be a valid, reliable and sensitive measure to assess the HRQoL in southern Chinese AIS patients. The EQ-5D-5L utility score showed good and significant correlations with SRS-22r total and all domain scores except for Satisfaction with Management.[16] Therefore this study aims to establish a prediction model that maps spine-specific scores represented by the SRS-22r, onto the EQ-5D-5L utility scores in AIS patients, with adjustment for demographic and clinical characteristics. We hypothesized that the Satisfaction with Management domain will not be significant predictor of the EQ-5D-5L score because such domain is not conceptually included in any of the five domains of EQ-5D-5L.

## Materials and methods

### Subjects

A prospective study with recruitment of southern Chinese AIS patients managed at a tertiary referral center for scoliosis during the months of August to October 2015 was performed. Exclusion criteria included patients with non-idiopathic scoliosis, those who could not understand traditional Chinese, with intellectual/ physical disability, or refused to participate. Ethics approval was obtained from the local Institutional Review Board of the University of Hong Kong / Hospital Authority Hong Kong West Cluster (HKWC/HKU IRB). Written informed consent was obtained from all recruited patients and the consent document was approved by the ethics committee. The reporting of this mapping study complied with the Mapping onto Preference-based measures reporting Standards (MAPS) statement.[17]

All eligible patients were approached by trained research personnel who distributed and allowed self-completion of both the EQ-5D-5L (Hong Kong (traditional Chinese) EQ-5D-5L Version 1.0, EuroQol)[18] and SRS-22r questionnaires.[19] To control for the ordering effect of completing the questionnaire, half of the patients were provided with EQ-5D-5L followed by SRS-22r, and the remaining half were given the questionnaires in the reversed order. All questionnaires were completed by the patients themselves.

During the visit, patients’ consultation and radiographic examinations were performed as usual. A spine surgeon measured the Cobb angle[20] on the standing whole spine posteroanterior radiograph taken at that appointment without any knowledge of this study. The curvatures were classified subsequently using the modified Lenke classification system,[21] which included six curve types: type 1 (main thoracic), type 2 (double thoracic), type 3 (double major; thoracic curve larger than lumbar curve), type 4 (triple major), type 5 (thoracolumbar or lumbar curve), type 6 (double major; thoracolumbar or lumbar curve larger than thoracic curve), and curvature magnitude (≤40° vs >40°) was noted. Clinical parameters also included treatment modalities of whether the patient had bracing or surgery, and the duration of bracing (<1 year vs ≥1 year) if applicable. Demographic data such as age and sex were collected.

### Study instruments

#### Refined Scoliosis Research Society 22-item (SRS-22r)

The SRS-22r questionnaire, a refinement of the SRS-24 questionnaire, was a disease-specific instrument used routinely during management of scoliosis patients. It had been previously validated in the Hong Kong Chinese scoliosis population.[19] It contained 22 items contributing to five main domains: Function (5 items), Pain (5 items), Self-image/appearance (5 items), Mental Health (5 items), Satisfaction with Treatment (Current/Previously performed—2 items).

#### EuroQoL 5-dimension 5-level (EQ-5D-5L)

The EQ-5D-5L is the latest version of the EQ-5D multi-attribute health classification system for measuring HRQOL and utility scores, consisting of five dimensions: mobility, self-care, usual activity, pain/discomfort and anxiety/depression. Each dimension had five severity levels: no problem, mild problem, moderate problems, severe problems, extreme problems. In principle, the utility scores are preference weights measured on a cardinal scale of 0–1, where ‘0’ indicates death and 1 indicates perfect health. Health states worse than death takes negative value of utility. Since the Chinese-specific EQ-5D-5L value set was currently unavailable, the EQ-5D-5L utility score was not determined through direct valuation of EQ-5D-5L health states. Rather, the two-step indirect approach was applied in this study to estimate EQ-5D-5L values applicable for Chinese population, as adopted in previous studies.[22, 23] The first step was the application of an indirect interim mapping method from a six-country study coordinated by EuroQoL group.[24] The EQ-5D-5L health status was transformed to EQ-5D-3L health status following the transition probabilities between the response patterns of EQ-5D-5L and EQ-5D-3L. Finally, EQ-5D-3L health status were scored according to a recently developed Chinese-specific EQ-5D-3L value set ranging from -0.149 for the worst health status (‘33333’) to 1 for the full health (‘11111’).[25]

### Statistical analysis

#### Model specification

Three separate models **(Table 1)** mapping SRS-22r onto EQ-5D-5L utility score were estimated by ordinary least squares (OLS) method. EQ-5D-5L scores were regressed on 1) five main terms (Function / Pain / Appearance / Mental Health / Satisfaction with Treatment) of SRS-22r subscale scores (Model 1), 2) as per 1 plus squared terms of subscale scores selected in Model 1 (Model 2), and 3) as per 2 plus demographic and clinical characteristics such as Cobb angle and treatment modalities (Model 3). Squared terms were added to the mapping function to account for possible non-linear association between SRS-22r and EQ-5D-5L utility score. The OLS method was the commonest estimation method, accounting for 80% of studies mapping HRQOL scores onto EQ-5D scores[26]. Backward stepwise selection approach was used to select an array of variables in each model. The F-test was used to retain variables with an exclusion criterion of P-value greater than 0.10. There was a total of three mapping models developed for researchers to decide which mapping model was chosen depending upon the availability of demographic and clinical data.

**Table 1:** Mapping models for EQ-5D-5L scores predicting from SRS-22r domain scores.

Model	Independent Variables	Estimation
1	SRS-22r Domain scores (Main effects)	OLS
2	SRS-22r Domain scores (Main effects and squared terms)	OLS
3	SRS-22r Domain scores (Main effects and squared terms); Clinical and demographic characteristics	OLS

Note: EQ-5D-5L = EuroQoL 5-dimension 5-level; SRS-22r = Refined Scoliosis Research Society-22; OLS = Ordinary Least Squares

#### Model validation and comparison

Model validation of three mapping functions were assessed by using the 10-fold cross-validation procedure.[27, 28] In brief, our sample is randomly partitioned into 10 equally sized subsamples. Of the 10 subsamples, one subsample and nine remaining subsamples were used as training and validation data, respectively. The cross-validation procedure was repeated 10 times with each of the nine subsamples (204–205 samples each) used once as the validation data. The model parameters, goodness-of-fit and predictive performances from the 10 folds were averaged to produce a single estimation.

Model goodness-of-fit was assessed using R-square, adjusted R-square, Akaike information criteria (AIC), and Bayesian information criteria (BIC) statistics. A better model goodness-of-fit was indicated if the R-square and adjusted R-square were higher; and the AIC and BIC values were lower. To assess the predictive performance of models, the differences between the predicted and observed EQ-5D-5L scores at individual level were examined by computing root mean square error (RMSE) and mean absolute error (MAE). The number of observations and the corresponding proportions in the sample where the absolute error (AE) was greater than 0.05 and 0.10, respectively, were calculated. The ranges of the achievable EQ-5D-5L scores from the resulting mapping models were compared with the theoretical range of the EQ-5D-5L scores computed according to Chinese-specific tariff.

All statistical analyses were conducted using the STATA software (StataCorp LP, College Station, Tex) version 13.0.

## Results

A total of 227 AIS patients were recruited. A majority of patients were female (75%), with a mean age of 15.6 years (standard deviation (SD): 4.5). 33.1% of this studied population were undergoing bracing or had corrective surgery performed. The mean Cobb angle was 25.0 degrees (SD: 11.4 degrees) at recruitment (**Table 2**). Descriptive statistics of EQ-5D-5L utility scores and SRS-22r domain scores were found in **Table 3**. The mean EQ-5D-5L utility scores was 0.931 (SD: 0.113; range: 0.339–1.000).

**Table 2:** Descriptive statistics of demographic and clinical characteristics.

		Overall (n = 227)
**Age (years, Mean±SD)**		15.5 ± 3.8
**Sex**		
	Male	57 (25.1%)
	Female	170 (74.9%)
**Cobb angle (degree, Mean±SD)**		23.9 ± 10.3
	≤40°, Mild or moderate	205 (90.3%)
	>40°, Severe	22 (9.7%)
**Treatment modality**		
	Wearing bracing	54 (23.8%)
	Surgery	21 (9.3%)
**Duration of Bracing**		
	<1 year	20 (37.0%)
	≥1 year	34 (63.0%)
**Modified Lenke Classification**		
	Thoracic curve (Types 1/2)	86 (37.9%)
	Lumbar curve (Type 5)	38 (16.7%)
	Thoracic & Lumbar curve (Types 3/4/6)	103 (45.4%)

Note: SD = standard deviation

**Table 3:** Descriptive statistics of EuroQoL 5-dimension 5-level (EQ-5D-5L) scores and refined scoliosis research society-22 (SRS-22r) domain scores.

			Overall (n = 227)		
		Theoretical Range	Mean ± SD	95% C.I.	Observed Range
**Utility Scores**					
	EQ-5D-5L score	-0.149–1.000	0.931 ± 0.113	0.909–0.954	0.339–1.000
**SRS-22 Domain**					
	Function/activity	1.0–5.0	4.774 ± 0.421	4.692–4.857	2.6–5.0
	Pain	1.0–5.0	4.667 ± 0.441	4.580–4.753	1.8–5.0
	Appearance	1.0–5.0	3.935 ± 0.641	3.809–4.061	2.0–5.0
	Mental Health	1.0–5.0	4.420 ± 0.584	4.306–4.534	2.6–5.0
	Satisfaction with management	0.0–5.0	1.069 ± 1.805	0.715–1.423	0.0–5.0

Note: SD = standard deviation; C.I. = Confidence Interval

The OLS regression analysis of the models and those validation results were shown in **Table 4** and **Table 5**, respectively. Main effects of five domain scores of SRS-22r were the predictors of the first model (model 1) but ‘Satisfaction’ domain score was dropped due to statistical insignificance (P-value>0.01). In model 2, the squared terms of four remaining domains were added for consideration but only the squared term of ‘Function/activity’ and ‘Pain’ domain scores (P-value = 0.041; P-value = 0.022) remained significant during the stepwise selection procedure. Cobb angle at recruitment (P-value<0.01) were retained in model 3 when adjusting for all demographic and clinical characteristics. By using ordinary least square estimation with clinical and demographic characteristics, EQ-5D-5L score was regressed on SRS-22r Domain scores with detailed formula equation of the selected model 3 found as expressed below:

$$\begin{array}{l} Predicted\;EQ-5D-5L\;score=\\ -0.366+0.489\;x\;\left(Function\;/\;activity\right)-0.042\;x\;{\left(Function\;/\;activity\right)}^{2}-0.221\;x\;\left(Pain\right)+0.031\;x\;{\left(Pain\right)}^{2}+0.023\;x\;\left(Appearance\right)+0.037\;x\;\left(Mental\;Health\right)+0.001\;x\;\left(Cobb\;angle\right) \end{array}$$

**Table 4:** Mapping models for patients with adolescent idiopathic scoliosis using main effects, squared terms of SRS-22r and patients' characteristics.

	Mapping Model (n = 227)					
	Main effects Model 1		Squared terms added Model 2		Clinical and demographic characteristics added Model 3	
SRS-22r Domain	Coeff.	(95% C.I)	Coeff.	(95% C.I)	Coeff.	(95% C.I)
Constant	-0.094	(-0.212,0.023)	-0.474	(-1.158,0.210)	-0.366	(-1.040,0.308)
Function / activity	0.119	(0.087,0.151)	0.559	(0.140,0.979)	0.489	(0.075,0.903)
Pain	0.046	(0.017,0.074)	-0.222	(-0.449,0.005)	-0.221	(-0.443,0.002)
Appearance	0.020	(0.001,0.040)	0.020	(0.001,0.039)	0.023	(0.005,0.042)
Mental Health	0.037	(0.015,0.059)	0.035	(0.013,0.057)	0.037	(0.016,0.058)
(Function / activity)^2^			-0.050	(-0.097,-0.002)	-0.042	(-0.089,0.005)
Pain^2^			0.031	(0.005,0.057)	0.031	(0.005,0.057)
Cobb angle					0.001	(0.001,0.002)
Goodness-of-fit						
*R*^*2*^	59.3%		60.4%		62.1%	
Adj *R*^*2*^	58.6%		59.3%		60.9%	
AIC	-536.647		-538.734		-546.650	
BIC	-519.567		-514.822		-519.321	
Predictive performance						
RMSE	0.073		0.072		0.071	
MAE	0.053		0.052		0.052	
AE > 0.05	40.9%		39.1%		39.1%	
AE > 0.10	18.7%		16.4%		15.1%	

Note: SRS-22r = Refined Scoliosis Research Society-22; AIC = Akaike information criteria; BIC = Bayesian information criteria; RMSE = root mean square error; MAE = mean absolute error; AE = absolute error; C.I. = Confidence Interval; Coeff. = Coefficient

**Table 5:** Predictive performance of three models in 10-fold cross-validation.

	Mapping Models for EQ-5D-5L scores		
	Model 1	Model 2	Model 3
Predictive performance			
RMSE	0.065	0.062	0.063
MAE	0.044	0.039	0.039
AE > 0.05	32.8%	30.7%	30.3%
AE > 0.10	13.8%	11.7%	11.7%

Note: EQ-5D-5L = EuroQoL 5-dimension 5-level; RMSE = root mean square error; MAE = mean absolute error; AE = absolute error

**Fig 1:**
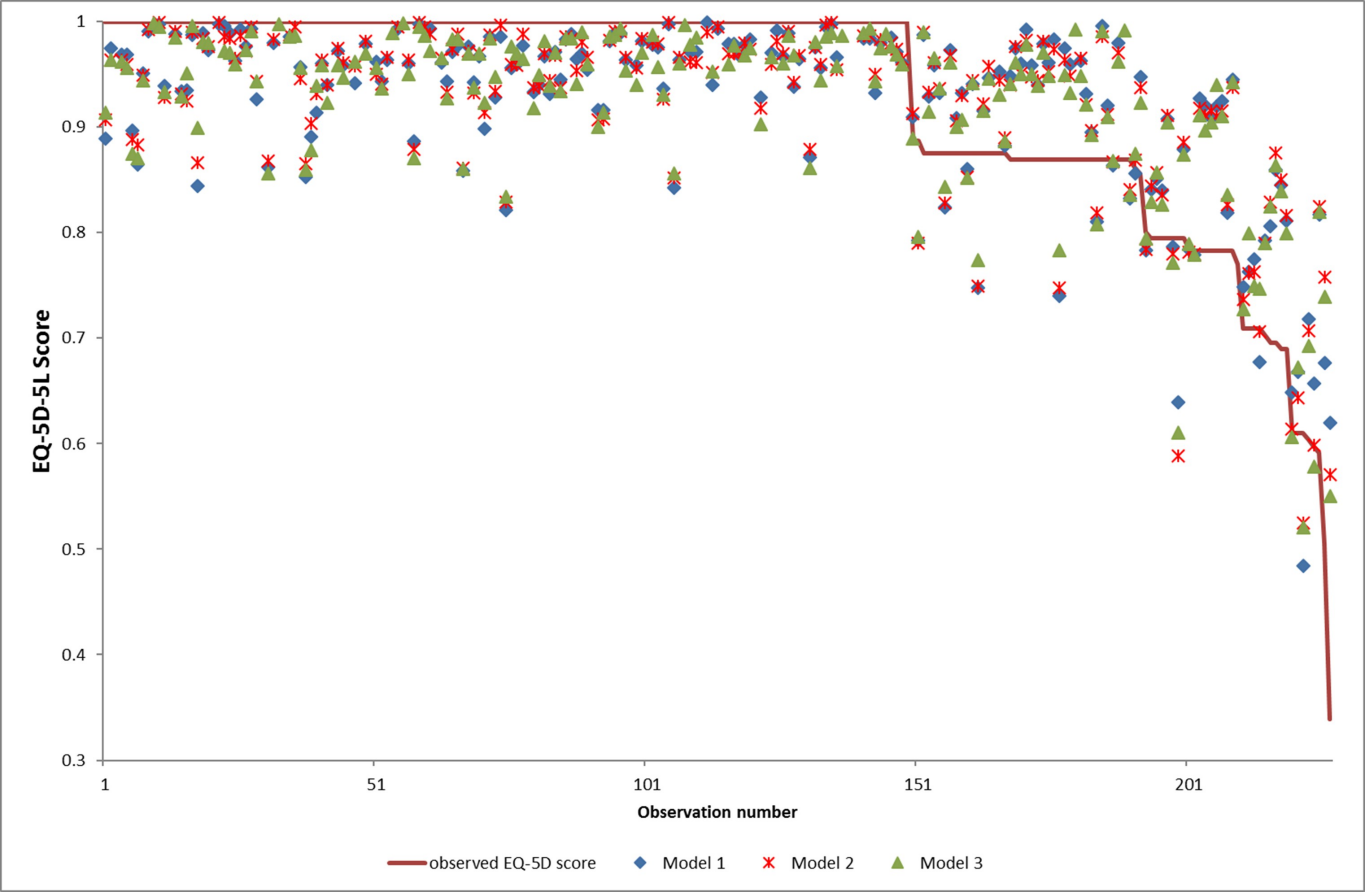
The scatterplot of observed and predicted EQ-5D-5L utility scores by mapping function. There was overestimation for more severe health states when the observed EQ-5D-5L utility scores was smaller than 0.8. Underestimation was demonstrated for observed EQ-5D-5L utility scores beyond 0.8.

Model 2 and 3 got R-square of 60% or above, demonstrating acceptable goodness-of-fit. The R-square, adjusted R-square, AIC and BIC values were further improved and optimized with adjustment of patients’ characteristics. Model 3 had better goodness-of-fit than Model 1 and 2. Predictive performance was considered good according to the error analysis and excellent values as expressed in terms of RMSE and MAE (**Table 4)**. The scatterplot of observed and predicted EQ-5D-5L scores by mapping function was shown in **Fig 1**. A pattern of overestimation was recognized for observed scores lower than 0.8, whereas underestimation was recognized for observed scores beyond 0.8. **Table 5** depicted the predictive performance of three models in the 10-fold cross-validation. In error analysis using cross-validation, RMSE and MAE were between 0.063–0.065 and between 0.039–0.044, respectively. Validation results of applying mapping models to AIS patients ascertained that models predicted the EQ-5D-5L scores accurately. Therefore, model 3 was recommended out of three mapping models given that the SRS-22r subscale scores and Cobb angle were available.

## Discussion

AIS is the most common pediatric spinal deformity affecting both physical and mental health. Treatment options involving observation, bracing and surgeries can impose various degrees of burden onto patients.[5, 29–33] Such burdens can be induced by a concern of curvature progression depending on its initial magnitude and patients’ maturity status, aesthetic concern, inconvenience of bracing during daily activities, as well as postoperative pain, and recovery for surgical patients.[34, 35] It is demonstrated that AIS patients may experience psychosocial difficulties, especially those undergoing active treatment, as compared to adults with scoliosis, who generally display fewer psychological problems.[35] Hence the varied quality of life of AIS patients and their treatment outcome warrant an assessment, especially in terms of economic evaluation in relation to different treatment options.

The SRS-22r questionnaire, being a disease-specific measure, is able to assess states and concerns of this particular diagnostic groups, and may have more items concerning functions most relevant to the disease.[36] However, such an instrument does not give the utility score for comparison across different disease populations. Health state utility values are usually based on generic instruments that permit comparisons between patient groups.[37] Therefore, it is desirable to have an equivalent and widely used generic instrument, which is shown to be successful in capturing the spine-specific questionnaire responses from AIS patients. The EQ-5D is recommended and widely used in economic evaluation as required by the NICE.[38] It has been most widely applied in recent years[39] as the EQ-5D is cognitively simple for self-completion, and it is found to be the most commonly used instrument in most cost-utility analysis studies conducted alongside clinical trials.[40] NICE states that ‘when EQ-5D data are not available or are inappropriate for the condition or effects of treatment, the valuation methods should be fully described and comparable to those used for EQ-5D’.[38] In particular, EQ-5D-5L was shown to have improved measurement properties and discriminatory power with reduced ceiling effects as compared to EQ-5D-3L.[41] Hence, the mapping of SRS-22r responses onto EQ-5D-5L score is required to enable an economic evaluation of treatment outcomes of AIS patients.[42]

Regarding the pattern of overestimation for observed scores lower than 0.8, and underestimation for observed scores beyond 0.8, there is a lower likelihood of being overestimated by this mapping model because of ceiling effects of the EQ-5D-5L utility score. About two-third (66%) respondents had a perfect health state of EQ-5D-5L, and thus those utility scores with maximum score of 1.0 are impossible to be overestimated.

The development of our mapping model not only aims to establish statistical relationship between EQ-5D-5L utility scores and scoliosis-specific SRS-22r domain scores, it also explores the possibility of developing accurate but simple-to-use mapping models leveraging conversion from existing SRS-22r data onto EQ-5D utility scores. It is mandatory to further investigate the accuracy and reliability of such mapping models in their predictions for AIS, as well as to ascertain the models’ suitability for this particular scoliotic group. SRS-22r dimension scores therefore are mapped onto EQ-5D-5L scores using a number of different model specifications. The predictive performance and goodness-of-fit indices of mapping models for AIS are evaluated on a variety of treatment modalities, regardless of active observation, bracing or surgical intervention.

There are three models developed in our attempt to seek the ability of mapping SRS-22r onto EQ-5D-5L at its best extent. Notably, through the inclusion of not only the demographic profiles, but with the four out of five possible SRS domain scores captured (Function/activity, Pain, Self-image/Appearance, and Mental Health except Satisfaction with Treatment), the model has improved from the base model to demonstrate an acceptable goodness-of-fit. By further addition of socio-demographic characteristics like sex and age of patients, and explanatory variables such as Cobb angle at the time of presentation and treatment status, the final model continues to offer improved model performance with good predictive performance and a relatively constant MAE. The clinical relevance of this developed mapping model should be emphasized, as the Cobb angle at the time of visit, the squared terms of ‘Function/activity’ and ‘Pain’ domain scores were found to be significant. These are variables which clinicians can professionally assess and can find their relevance. Addition of squared terms of SRS-22r domains provided evidence on the non-linear associations between spine-specific HRQOL scores and health utility score. On the other hand, despite not being found at a significant level statistically, aspects like patients’ perceived appearance and mental health may not be easily gauged by clinicians, but they were successfully included in the development of this mapping model.

The main limitation of this study relates to the Hong Kong value sets of EQ-5D-5L currently not being available until further social tariff of EQ-5D is developed. As this study being the first to map between EQ-5D-5L and SRS-22r questionnaires and focused specifically onto the local Chinese scoliosis population, no existing mapping function is available for comparison, and the mapping models generated can be population-specific. It will be ideal to have comparative studies in other countries or ethnic groups, and variable health-care systems to further validate our findings. A larger scale multi-center study of Chinese scoliosis patients can be helpful to provide large sample sizes for further testing of the developed model. Also, it is worth mentioning that the domain of treatment satisfaction of SRS-22r is not covered by EQ-5D-5L. Self-image, as a disease-specific domain, is not included either in the EQ-5D-5L. However, the addition of clinical parameter based on Cobb angle is accounted for, hence the model is only slightly undermined with good resultant predictive performance and goodness of fit still.

Nonetheless, this is the first study to establish statistical models mapping a scoliosis-specific HRQoL questionnaire onto a widely used generic utility score specifically for AIS patients. Although there are currently no standards or thresholds of whether our mapping models are adequately performed, they out-performed most of the published mapping models[43] in terms of goodness-of-fit indices and predictive performance. The mapping process has been perfected by incorporating scoliosis-specific clinical and demographic characteristics into the model. Not only is the achieved mapping model feasible to be used in economic evaluation of clinical research projects, this valid model has provided the basis for the ultimate assessment of QALYs, a measure of health benefit enabling a standardized approach for comparing economic evaluations across different healthcare areas.[13, 39]

## Conclusion

Being able to employ EQ-5L-5D through mapping based on existing SRS-22r data allows comparison of AIS with other populations or disease groups, and more importantly enables health economic appraisal for AIS patients, in terms of cost-utility of different treatment options, patients’ quality of life resulting from treatment undergone and their QALY. As the impact of a treatment on patients’ HRQoL and the impact on the length of life can both be encapsulated by QALY, a summary measure of health outcome can then be derived. Based on such health outcome, this will bring current assessment to a new phase in the aspect of clinical appraisal of different scoliosis centers, and orientate their management approach towards more effective use of resources. This can alter healthcare resource allocation decisions and can potentially reform healthcare policy on its largest scale. Moreover, this can bring a new perspective to the decision making on individual treatment option and management of AIS patients in the future.

## Supporting information

S1 Dataset Dataset used to generate the results of this study. (XLSX)
